# *In Vitro* and *In Vivo* Evaluation of the Antifungal Activity of APX001A/APX001 against Candida auris

**DOI:** 10.1128/AAC.02319-17

**Published:** 2018-02-23

**Authors:** Christopher L. Hager, Emily L. Larkin, Lisa Long, Fatima Zohra Abidi, Karen J. Shaw, Mahmoud A. Ghannoum

**Affiliations:** a Center for Medical Mycology, University Hospitals Cleveland Medical Center and Case Western Reserve University, Cleveland, Ohio, USA; b Amplyx Pharmaceuticals, San Diego, California, USA

**Keywords:** APX001, APX001A, susceptibility testing, yeast, efficacy, infection model, brain penetration, survival, Candida auris, antifungal susceptibility testing

## Abstract

Candida auris is an emerging multidrug-resistant yeast that has been responsible for invasive infections associated with high morbidity and mortality. C. auris strains often demonstrate high fluconazole and amphotericin B MIC values, and some strains are resistant to all three major antifungal classes. We evaluated the susceptibility of 16 C. auris clinical strains, isolated from a wide geographical area, to 10 antifungal agents, including APX001A, a novel agent that inhibits the fungal protein Gwt1 (glycosylphosphatidylinositol-anchored wall transfer protein 1). APX001A demonstrated significantly lower MIC_50_ and MIC_90_ values (0.004 and 0.031 μg/ml, respectively) than all other agents tested. The efficacy of the prodrug APX001 was evaluated in an immunocompromised murine model of disseminated C. auris infection. Significant efficacy (80 to 100% survival) was observed in all three APX001 treatment groups versus 50% survival for the anidulafungin treatment group. In addition, APX001 showed a significant log reduction in CFU counts in kidney, lung, and brain tissue (1.03 to 1.83) versus the vehicle control. Anidulafungin also showed a significant log reduction in CFU in the kidneys and lungs (1.5 and 1.62, respectively) but did not impact brain CFU. These data support further clinical evaluation of this new antifungal agent.

## INTRODUCTION

Candida auris is an emerging multidrug-resistant fungal pathogen associated with therapeutic drug failure and mortality (1). In a recent study, the mortality rate of patients with C. auris infections was 59%, with patients dying either with or as a result of the C. auris infection (2). A contributing factor is widespread resistance to fluconazole, as well as variable susceptibilities to amphotericin B, echinocandins, and other azoles. Examination of this collection of 54 isolates from Pakistan, India, South Africa, and Venezuela showed that the isolates were resistant to fluconazole (93%), voriconazole (54%), amphotericin B (35%), echinocandins (7%), and flucytosine (6%) using stringent breakpoints inferred from other Candida species (2). Resistance to echinocandins, the first-line treatment for Candida infections, is particularly problematic. In that same study, two of the isolates were triply resistant to azoles, echinocandins, and amphotericin B, resulting in a dearth of treatment options among the three approved major drug classes. C. auris can persist in the environment for at least 2 weeks (3), and patients with C. auris have been colonized for several months after the infection has resolved (1). This has led to environmental spread and transmission within health care facilities that are difficult to decontaminate and outbreaks that are difficult to treat (2, 4).

Contributing to the health threat is that C. auris is often misdiagnosed as Candida haemulonii, Candida famata, or Rhodotorula glutinis and cannot be accurately identified using commercial yeast identification systems such as Vitek 2 or API 20C AUX. Instead, diagnosis relies upon matrix-assisted laser desorption ionization–time of flight (MALDI-TOF) or molecular methods based upon sequencing the D1-D2 region of the 28s ribosomal DNA (rDNA) or the internal transcribed region of rDNA (1). This is problematic since many laboratories do not have rapid access to these technologies.

C. auris was originally identified in a patient in Japan in 2009 (5). Since then, it appears to have emerged simultaneously in several countries, resulting in the identification of four distinct clades (2). International travel has expedited the spread of this species, which has now been identified in numerous countries around the globe, including Japan, South Korea, South Africa, Kuwait, Kenya, United Kingdom, India, Pakistan, Colombia, Venezuela, and the United States (2, 6–9).

APX001 (formerly E1211) is a first-in-class small-molecule antifungal drug candidate. It is a water-soluble phosphate prodrug that is rapidly metabolized by systemic phosphatases to the active moiety, APX001A (formerly E1210). APX001A has a novel mechanism of action that is different than the other five classes of antifungal agents. APX001A targets the highly conserved fungal enzyme Gwt1. This conserved enzyme catalyzes the glycosylphosphatidylinositol (GPI) posttranslational modification that anchors eukaryotic cell surface proteins to the cell membrane (10, 11). In yeasts, GPI mediates cross-linking of cell wall mannoproteins to β-1,6-glucan. Inhibition of this enzyme in both Candida albicans and Saccharomyces cerevisiae results in inhibition of maturation and localization of GPI-anchored mannoproteins. Inhibiting Gwt1 blocks the inositol acylation step during synthesis of GPI-anchored proteins of the fungal cell wall, which compromises cell wall integrity, biofilm formation, and germ tube formation and produces severe fungal growth defects (10, 12). The closest mammalian ortholog of Gwt1 is the PIG-W protein, which is not sensitive to inhibition by APX001A (10).

APX001A has broad *in vitro* activity against major fungal pathogens, including Candida, Cryptococcus, Aspergillus, Scedosporium, Fusarium, and members of the Mucorales order (13–17). It maintains its activity against azole-resistant and echinocandin-resistant strains, which is consistent with the distinct mechanism of action. In invasive fungal infection animal models, the administration of APX001 (or APX001A) resulted in high survival rates and reduced colony counts of fungi in the lungs, kidneys, and brains of infected mice (18–21). The latter is particularly important given the brain dissemination associated with several invasive fungal infections (22).

In this study, we evaluated the activities of APX001A and APX001 against a panel of 16 diverse C. auris isolates and in a disseminated candidiasis infection model, respectively. These isolates were previously used to evaluate the activity of two compounds in clinical development, the glucan synthesis inhibitor SCY-078 and the echinocandin CD101 (23, 24). The strains included clinical isolates from Germany, Japan, South Korea, and India (23), and many demonstrated elevated MIC values for amphotericin B and fluconazole.

## RESULTS

### Antifungal susceptibility profile.

APX001A was highly active against all 16 strains of C. auris with markedly lower MIC_50_ and MIC_90_ values (concentrations that inhibit 50 and 90% of the tested isolates, respectively) than the other tested antifungals with an MIC_50_ of 0.004 μg/ml, an MIC_90_ of 0.031 μg/ml, and a range of values between 0.002 to 0.063 μg/ml (Table 1). These values are equal to or lower than those previously observed for the activity of APX001A against other Candida species (14, 16). The next most active agent was anidulafungin with MIC_50_ and MIC_90_ values of 0.125 and 0.25 μg/ml, respectively. Other compounds, including flucytosine, caspofungin, micafungin, and the three azoles (itraconazole, posaconazole, and voriconazole), demonstrated MIC_90_ values of 1 or 2 μg/ml. Amphotericin B was slightly less active, with MIC_90_ values of 4 or 8 μg/ml when read at 24 or 48 h, respectively (Table 1). The latter values are higher than the amphotericin B epidemiological cutoff of 2 μg/ml for the five other Candida species according to Clinical and Laboratory Standards Institute (CLSI) interpretive breakpoints, suggesting a trend for C. auris toward elevated MIC values or resistance (25). Fluconazole was the least active drug tested with both an MIC_50_ and MIC_90_ of >64 μg/ml, an observation consistent with widespread resistance that has been reported elsewhere (2).

**TABLE 1:** Summary of microbiological activity of APX001A and comparators against 16 strains of C. auris

Antifungal agent	Treatment period (h)	MIC (μg/ml)^*a*^		
		Range	MIC_50_	MIC_90_
APX001A	24	0.002–0.063	0.004	0.031
5FC	48	0.5–1	0.5	1
AFG	24	0.125–0.25	0.125	0.25
CAS	24	0.25–1	0.5	1
MFG	24	0.25–2	1	2
AMB	24	0.5–8	2	4
	48	2–8	4	8
FLC	24	1–>64	16	>64
	48	2–>64	>64	>64
ITC	48	<0.063–1	0.5	1
POS	48	0.25–1	0.25	1
VRC	24	<0.063–1	0.5	1
	48	<0.063–2	0.5	2

a MIC values were read at either 50 or 100% inhibition as indicated by CLSI; APX001A was read at 50% inhibition and at 24 h as previously determined.

### Survival in a disseminated C. auris infection model.

A neutropenic mouse model of disseminated candidiasis was developed to enable the *in vivo* assessment of the efficacy of antifungal compounds against C. auris. To determine the optimal inoculum that would cause a disseminated infection, immunocompromised mice (*n* = 5) were inoculated with 10^6^ to 10^8^ blastospores in 0.1 ml of phosphate-buffered saline (PBS) (via the tail vein) of clinical isolates CBS 12766 (India) and CBS 12777 (India). Survival was monitored as a marker of disease for 14 days. Based on these preliminary experiments, an inoculum of 3 × 10^7^ blastospores of CBS 12766 was found to be optimal for use in subsequent efficacy studies (unpublished observations).

APX001 intraperitoneal (i.p.) dosing regimens were chosen that included both twice daily (BID) and three times daily (TID) for two different dose levels (78 and 104 mg/kg). These dose levels were chosen based upon doses successfully used in a previous animal model (20). Phase 1 studies of APX001 has demonstrated that it has a long half-life in humans (∼2.5 days) (26, 27); however, its half-life in mice is short (1 to 1.8 h depending upon the route of administration), necessitating multiple daily doses (20, 28). The anidulafungin dose (10 mg/kg BID i.p. or 20 mg/kg/24 h) was chosen since it is above the dose associated with killing of 1 log CFU of several strains of C. albicans (1.95 to 13 mg/kg/24 h) and Candida glabrata (3.31 to 11.8 mg/kg/24 h), which were previously determined for anidulafungin in similar murine neutropenic disseminated candidiasis models (29).

**FIG 1:**
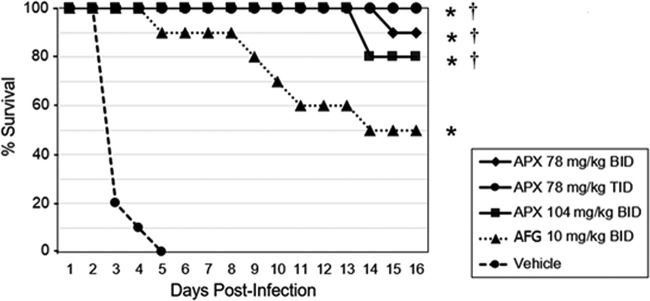
Survival curves in mice infected with C. auris CBS 12766. Treatments were administered by i.p. injection (*n* = 10/group): APX001 at doses of 78 mg/kg BID, 78 mg/kg TID, or 104 mg/kg BID; 10 mg/kg anidulafungin BID; or vehicle BID. Treatment began 2 h postinoculation and continued for 7 days. Survival was monitored until day 16. Survival was plotted by Kaplan-Meier analysis, and differences in the percent survival among groups were analyzed by the log-rank test and the Fisher exact test, respectively. Abbreviations: APX, APX001A; AFG, anidulafungin. *, *P* ≤ 0.0001 versus vehicle control; †, *P* ≤ 0.05 versus anidulafungin.

In this disseminated C. auris infection model, 100% mortality was observed for the vehicle BID-treated control group by day 5 (Fig. 1). The percent survivals on day 16 for mice in the APX001 78-mg/kg BID, 78-mg/kg TID, 104-mg/kg BID, and anidulafungin 10-mg/kg BID groups were 90, 100, 80, and 50%, respectively. Mice in all APX001 treatment groups had a significantly higher percent survival compared to the anidulafungin (*P* = 0.034) and vehicle groups (*P* < 0.0001). There was no statistically significant difference between the three APX001-treated groups. The anidulafungin treated mice also had a significantly higher percent survival compared to the vehicle group (*P* < 0.0001).

### Fungal burden: CFU reductions.

**FIG 2:**
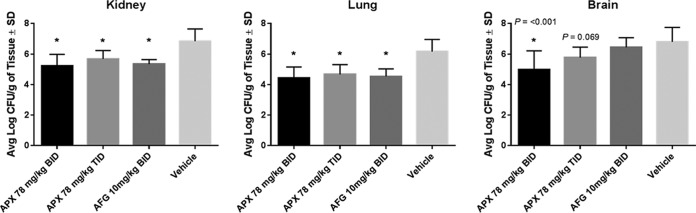
Reduction in fungal burden in the mouse kidneys, lungs, and brains at 48 h postinfection with C. auris CBS 12766. Treatments were administered by i.p. injection (*n* = 5/group): APX001 at doses of 78 mg/kg BID or 78 mg/kg TID; 10 mg/kg anidulafungin BID; or vehicle control BID. Treatment began at 2 h postinoculation and continued for 2 days. Animals were sacrificed at 48 h, and the fungal burden was determined. Differences in mean CFU in mouse kidneys, lungs, and brains were compared to the vehicle control using a one-way ANOVA with a post hoc Tukey test. Abbreviations: APX, APX001; AFG, anidulafungin. *, *P* ≤ 0.001 versus control.

In a subsequent study of disseminated C. auris infection, mice were sacrificed at 48 h for determination of the lung, kidney, and brain fungal burdens after i.p. dosing of APX001 (78 mg/kg BID, 78 mg/kg TID) and anidulafungin (10 mg/kg BID) versus the vehicle control. The 48-h time point was chosen to ensure 100% survival at the time of sacrifice. Both doses of APX001 and anidulafungin demonstrated a significant log_10_ reduction in kidney CFU/g (1.16 to 1.62) and lung CFU/g (1.49 to 1.74) at 48 h versus the vehicle control group (*P* = 0.0003) (Fig. 2). There was no statistically significant difference in the reduction of fungal burden in lung and kidney for the two APX001-treated groups.

Since Candida infections can be hematogenously disseminated to other organs including the brain, CFU counts were also enumerated from brain tissue at 48 h. The APX001 78-mg/kg BID treatment group demonstrated a 1.83 and 1.48 log reductions compared to both the vehicle control and the anidulafungin-treated group (*P* = 0.0004 and 0.005, respectively). The APX001 78-mg/kg BID and TID treatment groups were not statistically different from each other, although the APX001 TID treatment group did not reach statistical significance versus the vehicle control (*P* = 0.0691).

At the 48-h time point, APX001 demonstrated a significant reduction in the kidney, lung, and brain fungal burdens, whereas anidulafungin demonstrated a significant reduction in only kidney and lung fungal burdens (Fig. 2). These findings are consistent with poor central nervous system (CNS) penetration observed for the echinocandins (30).

## DISCUSSION

The Centers for Disease Control (CDC) has identified C. auris as a serious global health threat that can cause serious invasive infections (https://www.cdc.gov/fungal/diseases/candidiasis/candida-auris-qanda.html). C. auris is often multidrug resistant, is difficult to detect, and has caused outbreaks in health care settings. As a result, new treatment options are required to combat this emerging health care threat, especially for multidrug-resistant strains. In this study, we evaluated the *in vitro* activity of APX001A against a panel of 16 diverse C. auris isolates, as well as the *in vivo* activity of the prodrug APX001, in a disseminated candidiasis model that utilized one of the isolates that originated from India (CBS 12766).

APX001A demonstrated an MIC_90_ value that was 8-fold lower than the next most active drug, anidulafungin, and >30-fold lower than all other compounds tested (Table 1). In addition to the improved C. auris microbiological activity versus currently marketed drugs, APX001A also demonstrated a lower MIC_90_ than two other agents in clinical development, SCY-078 and CD101 that were evaluated versus the same 16 strains; the MIC_90_s for SCY-078 and CD101 were 1 and 0.25 μg/ml, respectively (23, 24). Recently, other compounds in development have been evaluated against a panel of 100 strains housed at the CDC representing the four C. auris clades that included triply resistant isolates. Against this panel, the MIC_90_ values for VT-1598, a fungal CYP51 inhibitor, and SCY-078 were both 1 μg/ml (31, 32).

APX001 was evaluated in a disseminated C. auris infection model in immunocompromised mice. The echinocandin anidulafungin was chosen as a control since it was the second most microbiologically active agent and represents the class of agents most likely to be used as first-line therapy (1, 9). The dose of anidulafungin that was chosen has been previously shown in pharmacokinetic/pharmacodynamic modeling experiments to be higher than both the stasis dose and the dose resulting in 1 log killing of C. albicans and C. glabrata (29). Thus, it was anticipated that this dose regimen would be effective in this model. The doses of APX001 used in this model are greater than or equal to doses that have been previously shown to be effective in animal models of candidiasis or other invasive fungal infections (18, 20, 28) and achieve area under the concentration-time curve (AUC) after 24 h values in mice that are severalfold (∼3) lower than the steady-state AUC_0−24_ values anticipated to be used in phase 2 studies in humans (26, 27). In the current model of disseminated C. auris infection, APX001 resulted in significantly better survival than anidulafungin. Both APX001 and anidulafungin demonstrated significant and equivalent decreases in kidney and lung CFU at 48 h. However, only APX001 demonstrated a reduction in brain CFU, a finding consistent with the brain penetration observed in ^14^C-APX001 distribution studies where radioactivity was detected in tissues associated with invasive fungal infections (33). This contrasts with the poor CNS penetration observed for the echinocandins (30). It is possible that better brain penetration (or other unexamined compartments that show a differentiated distribution pattern such as eye) may have contributed to better overall survival in this model.

One limitation of this study is that only APX001 78-mg/kg BID dosing—and not the 78-mg/kg TID dosing—demonstrated a statistically significant reduction in brain CFU. It is possible that larger numbers of animals in each treatment group would allow resolution of this discrepancy, or alternatively, evaluating fungal brain burden at a time point greater than 48 h. In addition, the efficacy of APX001 administration in a delayed therapy model requires further investigation.

We demonstrated here that APX001A and its prodrug form, APX001, have potent activity against C. auris both *in vitro* and *in vivo*, respectively. APX001 may be a viable treatment option for infections attributable to the emerging nosocomial pathogen C. auris, especially for infections due to strains that are resistant to multiple drug classes.

## MATERIALS AND METHODS

### Isolates tested.

A total of 16 clinical isolates of C. auris from a wide geographic area (Germany, Japan, South Korea, and India) were tested for their susceptibility to 10 different antifungal agents. Fourteen of these C. auris isolates were obtained from the CBS-KNAW Fungal Biodiversity Centre, Utrecht, the Netherlands (CBS strains 10913, 12372, 12373, 12766, 2768, and 12770 to 12777), and the other two strains were from the Center for Medical Mycology Culture Collection characterized previously (23, 24). Candida krusei ATCC 6258 and Candida parapsilosis ATCC 22019 were used as quality control strains in susceptibility testing. One of the clinical isolates of C. auris (CBS 12766, isolated in India) was used as the infecting strain in the animal model of disseminated infection.

### Antifungal agents.

The activities of 10 antifungals against C. auris were tested, including anidulafungin (Pfizer Pharmaceuticals, New York, NY), caspofungin (Merck Co., Kenilworth, NJ), flucytosine (Astellas Pharma US, Inc., Northbrook, IL), micafungin (Astellas Pharma, Inc., Tokyo, Japan), and amphotericin B, fluconazole, itraconazole, posaconazole, and voriconazole (AMB, FLC, ITC, POS, and VRC were obtained from Sigma-Aldrich, St. Louis, MO). APX001A was originally synthesized at Eisai Co., Ltd., Tokyo, Japan, and obtained from Amplyx Pharmaceuticals.

### Antifungal susceptibility testing.

To establish the antimicrobial activity of APX001A against 16 strains of C. auris, broth microdilution susceptibility testing was performed according to the guidelines in the CLSI standard M27-A3 (34). Similar to the echinocandins, the APX001A MICs were evaluated after 24 h of incubation and read as 50% inhibition as previously described (16). MIC values were evaluated visually at 24 and 48 h, and endpoints were recorded at the lowest drug concentration to inhibit 50 or 100% of growth compared to the growth control (34).

### Animal use and care.

All animal-related study procedures were compliant with the Animal Welfare Act, the *Guide for the Care and Use of Laboratory Animals*, and the Office of Laboratory Animal Welfare. Mice were used upon review and approval of an addendum to an existing protocol by the Animal Care and Use Committee of Case Western Reserve University. Female CD1 mice (Charles River Laboratories, Wilmington, MA) with a body weight of ∼25 g were obtained and allowed to acclimate for a minimum of 5 days prior to use. Environmental controls for the animal room were set to maintain a temperature of 16 to 22°C, a relative humidity of 30 to 70%, and a 12:12 hourly light-dark cycle.

### Disseminated C. auris infection model.

Mice were immunosuppressed by i.p. injection of cyclophosphamide at 200 mg/kg 3 days before and 150 mg/kg 1 day after challenge. On the day of the challenge, blood was collected from one mouse from each group for a white blood cell count to verify immunosuppression. Mice were infected via the tail vein with 3 × 10^7^ C. auris CBS 12766 blastospores in 0.1 ml of PBS. Treatment was initiated 2 h postchallenge with test compounds being administered by i.p. injection. Treatment groups consisted of (i) vehicle control, (ii) APX001 at 78 mg/kg BID, 78 mg/kg TID, and 104 mg/kg BID, and (iii) anidulafungin at 10 mg/kg BID. Survival was monitored for 16 days postinoculation. Differences in cohorts were analyzed by the log-rank test and the Fisher exact test.

In a subsequent experiment, mice were sacrificed (five animals per group) 48 h postinfection and their kidneys, lungs, and brains were harvested aseptically and weighed. Tissues were homogenized and serially diluted in PBS, with homogenates plated onto potato dextrose agar (Difco Laboratories), and then incubated for 48 h to determine the number of CFU. The tissue fungal burden was expressed as the average log CFU/g of tissue. Differences in mean CFU in kidneys, lungs, and brains were compared to the vehicle control using a one-way analysis of variance (ANOVA) with a post hoc Tukey test. A *P* value of < 0.05 was considered statistically significant.

## References

[1] Vallabhaneni S, Kallen A, Tsay S, Chow N, Welsh R, Kerins J, Kemble SK, Pacilli M, Black SR, Landon E, Ridgway J, Palmore TN, Zelzany A, Adams EH, Quinn M, Chaturvedi S, Greenko J, Fernandez R, Southwick K, Furuya EY, Calfee DP, Hamula C, Patel G, Barrett P, Lafaro P, Berkow EL, Moulton-Meissner H, Noble-Wang J, Fagan RP, Jackson BR, Lockhart SR, Litvintseva AP, Chiller TM 2016 Investigation of the first seven reported cases of *Candida auris*, a globally emerging invasive, multidrug-resistant fungus: United States, May 2013-August 2016. MMWR Morb Mortal Wkly Rep 65:1234–1237. doi:10.15585/mmwr.mm6544e1. 27832049

[2] Lockhart SR, Etienne KA, Vallabhaneni S, Farooqi J, Chowdhary A, Govender NP, Colombo AL, Calvo B, Cuomo CA, Desjardins CA, Berkow EL, Castanheira M, Magobo RE, Jabeen K, Asghar RJ, Meis JF, Jackson B, Chiller T, Litvintseva AP 2017 Simultaneous emergence of multidrug-resistant *Candida auris* on three continents confirmed by whole-genome sequencing and epidemiological analyses. Clin Infect Dis 64:134–140. doi:10.1093/cid/ciw691. 27988485 PMC5215215

[3] Piedrahita CT, Cadnum JL, Jencson AL, Shaikh AA, Ghannoum MA, Donskey CJ 2017 Environmental surfaces in healthcare facilities are a potential source for transmission of *Candida auris* and other *Candida* species. Infect Control Hosp Epidemiol 38:1107–1109. doi:10.1017/ice.2017.127. 28693657

[4] Welsh RM, Bentz ML, Shams A, Houston H, Lyons A, Rose LJ, Litvintseva AP 2017 Survival, persistence, and isolation of the emerging multidrug-resistant pathogenic yeast *Candida auris* on a plastic health care surface. J Clin Microbiol 55:2996–3005. doi:10.1128/JCM.00921-17. 28747370 PMC5625385

[5] Satoh K, Makimura K, Hasumi Y, Nishiyama Y, Uchida K, Yamaguchi H 2009 *Candida auris* sp. nov., a novel ascomycetous yeast isolated from the external ear canal of an inpatient in a Japanese hospital. Microbiol Immunol 53:41–44. doi:10.1111/j.1348-0421.2008.00083.x. 19161556

[6] Magobo RE, Corcoran C, Seetharam S, Govender NP 2014 *Candida auris*-associated candidemia, South Africa. Emerg Infect Dis 20:1250–1251. doi:10.3201/eid2007.131765. 24963796 PMC4073876

[7] Emara M, Ahmad S, Khan Z, Joseph L, Al-Obaid I, Purohit P, Bafna R 2015 *Candida auris* candidemia in Kuwait, 2014. Emerg Infect Dis 21:1091–1092. doi:10.3201/eid2106.150270. 25989098 PMC4451886

[8] Schelenz S, Hagen F, Rhodes JL, Abdolrasouli A, Chowdhary A, Hall A, Ryan L, Shackleton J, Trimlett R, Meis JF, Armstrong-James D, Fisher MC 2016 First hospital outbreak of the globally emerging *Candida auris* in a European hospital. Antimicrob Resist Infect Control 5:35. doi:10.1186/s13756-016-0132-5. 27777756 PMC5069812

[9] Sarma S, Upadhyay S 2017 Current perspective on emergence, diagnosis and drug resistance in *Candida auris*. Infect Drug Resist 10:155–165. doi:10.2147/IDR.S116229. 28652784 PMC5476417

[10] Watanabe NA, Miyazaki M, Horii T, Sagane K, Tsukahara K, Hata K 2012 E1210, a new broad-spectrum antifungal, suppresses *Candida albicans* hyphal growth through inhibition of glycosylphosphatidylinositol biosynthesis. Antimicrob Agents Chemother 56:960–971. doi:10.1128/AAC.00731-11. 22143530 PMC3264227

[11] Orlean P, Menon AK 2007 Thematic review series: lipid posttranslational modifications. GPI anchoring of protein in yeast and mammalian cells, or: how we learned to stop worrying and love glycophospholipids. J Lipid Res 48:993–1011. 17361015 10.1194/jlr.R700002-JLR200

[12] McLellan CA, Whitesell L, King OD, Lancaster AK, Mazitschek R, Lindquist S 2012 Inhibiting GPI anchor biosynthesis in fungi stresses the endoplasmic reticulum and enhances immunogenicity. ACS Chem Biol 7:1520–1528. doi:10.1021/cb300235m. 22724584

[13] Castanheira M, Duncanson FP, Diekema DJ, Guarro J, Jones RN, Pfaller MA 2012 Activities of E1210 and comparator agents tested by CLSI and EUCAST broth microdilution methods against *Fusarium* and *Scedosporium* species identified using molecular methods. Antimicrob Agents Chemother 56:352–357. doi:10.1128/AAC.05414-11. 22083469 PMC3256086

[14] Miyazaki M, Horii T, Hata K, Watanabe NA, Nakamoto K, Tanaka K, Shirotori S, Murai N, Inoue S, Matsukura M, Abe S, Yoshimatsu K, Asada M 2011 *In vitro* activity of E1210, a novel antifungal, against clinically important yeasts and molds. Antimicrob Agents Chemother 55:4652–4658. doi:10.1128/AAC.00291-11. 21825291 PMC3186989

[15] Pfaller MA, Duncanson F, Messer SA, Moet GJ, Jones RN, Castanheira M 2011 *In vitro* activity of a novel broad-spectrum antifungal, E1210, tested against *Aspergillus* spp. determined by CLSI and EUCAST broth microdilution methods. Antimicrob Agents Chemother 55:5155–5158. doi:10.1128/AAC.00570-11. 21844312 PMC3194992

[16] Pfaller MA, Hata K, Jones RN, Messer SA, Moet GJ, Castanheira M 2011 *In vitro* activity of a novel broad-spectrum antifungal, E1210, tested against *Candida* spp. as determined by CLSI broth microdilution method. Diagn Microbiol Infect Dis 71:167–170. doi:10.1016/j.diagmicrobio.2011.05.001. 21696907

[17] Pfaller MA, Watanabe N, Castanheira M, Messer SA, Jones RN 2011 Pre-clinical development of antifungal susceptibility test methods for the testing of the novel antifungal agent E1210 versus *Candida*: comparison of CLSI and European Committee on Antimicrobial Susceptibility Testing methods. J Antimicrob Chemother 66:2581–2584. doi:10.1093/jac/dkr342. 21873291

[18] Hata K, Horii T, Miyazaki M, Watanabe NA 2011 *In vitro* and in vivo antifungal activities of E1211, a water-soluble prodrug of E1210. Intersci Conf Antimicrob Agents Chemother, abstr F1-1377.

[19] Watanabe NA, Horii T, Miyazaki M, Hata K 2011 *In vitro* activity of E1210 and in vivo activity of E1211, a water-soluble prodrug of E1210, in combination with other antifungals. Intersci Conf Antimicrob Agents Chemother, abstr F1-1378.

[20] Gebremariam T, Alkhazraji S, Alqarihi A, Wiederhold NP, Shaw KJ, Patterson TF, Filler SG, Ibrahim A 2017 APX001 protects immunosuppressed mice from *Rhizopus delemar* infection. Abstr IDweek 2017, poster 1521.

[21] Schell WA, Giamberardino C, Shaw KJ, Perfect JR 2017 Efficacy of oral APX001 in a murine model of cryptococcal meningitis. Abstr IDweek 2017, poster 1529,

[22] Ueno N, Lodoen MB 2015 From the blood to the brain: avenues of eukaryotic pathogen dissemination to the central nervous system. Curr Opin Microbiol 26:53–59. doi:10.1016/j.mib.2015.05.006. 26048316 PMC10538213

[23] Larkin E, Hager C, Chandra J, Mukherjee PK, Retuerto M, Salem I, Long L, Isham N, Kovanda L, Borroto-Esoda K, Wring S, Angulo D, Ghannoum M 2017 The emerging pathogen *Candida auris*: growth phenotype, virulence factors, activity of antifungals, and effect of SCY-078, a novel glucan synthesis inhibitor, on growth morphology and biofilm formation. Antimicrob Agents Chemother 61:e02396-16. doi:10.1128/AAC.02396-16. 28223375 PMC5404565

[24] Larkin EL, Long L, Ghannoum MA 2017 Susceptibility of recent *Candida auris* isolates to the novel echinocandin CD101 and comparator antifungal agents. Abstr ECCMID 2017, poster 9037.

[25] CLSI. 2016 Epidemiological cutoff values for antifungal susceptibility testing, 1st ed, supplement M59. Clinical and Laboratory Standards Institute, Wayne, PA.

[26] Hodges MR, Ople E, Shaw KJ, Mansbach RS, van Marle S, van Hoogdalem E, Kramer W, Wedel P 2017 Phase 1 study to assess safety, tolerability and pharmacokinetics of single and multiple oral doses of APX001 and to investigate the effect of food on APX001 bioavailability. Abstr IDweek 2017, poster 1860.

[27] Hodges MR, Ople E, Shaw KJ, Mansbach RS, van Marle S, van Hoogdalem E, Wedel P, Kramer W 2017 First-in-human study to assess safety, tolerability and pharmacokinetics of APX001 administered by intravenous infusion to healthy subjects. Abstr IDweek 2017, poster 1840.

[28] Wiederhold NP, Najvar LK, Fothergill AW, McCarthy DI, Bocanegra R, Olivo M, Kirkpatrick WR, Everson MP, Duncanson FP, Patterson TF 2015 The investigational agent E1210 is effective in treatment of experimental invasive candidiasis caused by resistant *Candida albicans*. Antimicrob Agents Chemother 59:690–692. doi:10.1128/AAC.03944-14. 25331706 PMC4291422

[29] Andes D, Diekema DJ, Pfaller MA, Prince RA, Marchillo K, Ashbeck J, Hou J 2008 In vivo pharmacodynamic characterization of anidulafungin in a neutropenic murine candidiasis model. Antimicrob Agents Chemother 52:539–550. doi:10.1128/AAC.01061-07. 18070979 PMC2224754

[30] Chen SC, Slavin MA, Sorrell TC 2011 Echinocandin antifungal drugs in fungal infections: a comparison. Drugs 71:11–41. doi:10.2165/11585270-000000000-00000. 21175238

[31] Berkow EL, Le N, Peterson J, Garvey EP, Yates CM, Schotzinger RJ, Lockhart SR 2017 *In vitro* activity of a novel Cyp51 inhibitor, VT-1598, against clinical isolates of *Candida auris*. Abstr ASM Microbe, poster 304.

[32] Berkow EL, Angulo D, Lockhart SR 2017 *In vitro* activity of a novel glucan synthase inhibitor, SCY-078, against clinical isolates of *Candida auris*. Antimicrob Agents Chemother 61:e00435-17. doi:10.1128/AAC.00435-17. 28483955 PMC5487607

[33] Mansbach RS, Shaw KJ, Hodges MR, Coleman S, Fitzsimmons ME 2017 Absorption, distribution, and excretion of [14C]-APX001 after single-dose administration to rats and monkeys. Abstr IDweek 2017, poster 1513.

[34] CLSI. 2008 Reference method for broth dilution antifungal susceptibility testing of yeasts; approved standard, 3rd ed Document M27-A3. Clinical and Laboratory Standards Institute, Wayne, PA.

